# PD-L1 Expression Level Displays a Positive Correlation with Immune Response in Pancreatic Cancer

**DOI:** 10.1155/2020/8843146

**Published:** 2020-09-25

**Authors:** Lei Zhao, Yaming Cao

**Affiliations:** ^1^Department of Hepatobiliary and Pancreatic Surgery, First Hospital of China Medical University, Shenyang, Liaoning 110001, China; ^2^Department of Immunology, College of Basic Medical Sciences, China Medical University, Shenyang, Liaoning 110122, China

## Abstract

The expression of PD-L1 could be a novel biomarker which predicts that patients are more likely to respond to immunotherapy. Our study investigated the relationship among clinicopathological characteristics, prognosis, PD-L1 expression levels, and FOXP3^+^ Treg infiltration. In addition, the relationship among clinicopathological characteristics, prognosis, PD-L1 expression levels, and FOXP3^+^ Treg infiltration was explored. Furthermore, the relationship between PD-L1 expression and FOXP3^+^ Treg infiltration was examined. We found that 41.3% of pancreatic cancer patients had PD-L1-positive staining; both PD-L1 expression levels and FOXP3^+^ Treg infiltration were significantly associated with depth of invasion, lymph node metastasis, distant metastasis, and pTNM. In addition, PD-L1 expression and FOXP3^+^ Treg infiltration also could be prognostic biomarkers for pancreatic cancer.

## 1. Introduction

Pancreatic cancer, a common and highly lethal malignant tumor of the digestive tract, is one of the most aggressive cancers with a dismally low 5-year overall survival rate (<5%) [1]. Although pancreatic cancer ranks as the sixth cancer-related cause of death in China [2, 3], patients still have a poor survival rate because early diagnosis is difficult, and most patients are usually diagnosed at the advanced stage, thus losing the opportunity for surgery. In addition, the patients who undergo radical surgical resection often experience distant metastasis and recurrence. Furthermore, chemotherapy resistance is very common in patients with pancreatic cancer. Therefore, gene immunotherapy is an important area of research in the treatment of pancreatic cancer [4]. How to enhance the capacity of antitumor treatment in the tumor microenvironment is an important study topic.

Programmed death 1 (PD-1), which was initially discovered in 1992, is an immune checkpoint and expressed on the surface of activated T-cells after antigen recognition, such as activated CD4^+^ T-cells, CD8^+^ T-cells, and several other immune cells [5]. PD-1 is involved in the classic type of programmed cell death and has two ligand molecules, namely, PD-L1 and PD-L2 (also known as B7-H1 and B7-DC, respectively). Among the ligands, PD-L1 is closely associated with the tumor microenvironment and expressed by various cell types, including tumor cells, tumor-associated macrophages, bone marrow-derived inhibitory cells, dendritic cells, T-cells, and B-cells [6–8]. In previous studies, PD-L1 was shown to potentially cause tumor cells to evade antitumor immune responses by inducing T-cell apoptosis [9]. In several other studies, inhibition of the PD-1/PD-L1 pathway reportedly could lead to a stronger tumor regression in cellular immunotherapies [10]. Currently, anti-PD-1 and anti-PD-L1 immunotherapies have achieved satisfactory effects in melanoma, non-small-cell lung cancer, renal cancer, and Hodgkin's lymphoma [11, 12] and are promising therapeutic methods.

Regulatory T-cells (Tregs) are a subset of T lymphocytes and can inhibit the proliferation and cytokine secretion of effector T lymphocytes. In a previous study, depletion of Tregs increased antitumor immunity [13]. The PD-1/PD-L1 signaling pathway can promote the proliferation of Tregs by inhibiting the phosphorylation of mTOR and Akt [14]. The level of tumor-infiltrating Tregs in PD-L1-positive tumors is higher than that in PD-L1-negative tumors [15]. In addition, FOXP3 is a fork-head helical transcription factor and plays a major regulatory role in the development and control of Tregs [16]. The increased number of infiltrating FOXP3^+^ Tregs was shown to be highly correlated with the patient outcome in several cancers [17–19]. However, the opposite conclusion was reported in several studies regarding colorectal cancer (CRC) [20, 21].

In the present study, the PD-L1 expression levels and the FOXP3^+^ Treg infiltration were investigated in a cohort consisting of 63 pancreatic cancer patients. In addition, the relationship among clinicopathological characteristics, prognosis, PD-L1 expression levels, and FOXP3^+^ Treg infiltration was explored. Furthermore, the relationship between PD-L1 expression and FOXP3^+^ Treg infiltration was examined.

## 2. Materials and Methods

### 2.1. Tissue Microarray

The pancreatic cancer tissue microarrays (HPan-Ade120Sur-01), which consisted of formalin-fixed, paraffin-embedded (FFPE) human pancreatic cancer tissues (*n* = 63) and normal adjacent tissues (*n* = 57), were purchased from Outdo Biotech Company (Shanghai, China). The tissue samples were obtained from patients who underwent radical resection of pancreatic cancer between 2009 and 2010. The hematoxylin and eosin (H&E) staining results showed that all cancer tissue samples were positively stained.

### 2.2. Information of Pancreatic Cancer Patients

The study cohort consisted of 63 pancreatic cancer patients which included 41 males and 22 females; average age was 65.52 ± 9.51 years (range: 41–85 years). The follow-up period was from July 2009 to November 2014. At the last follow-up, 13 patients were alive. The average survival time was 21.8 ± 19.4 months (range: 1–65 months). Among surviving patients, 4 patients had liver metastasis and 5 patients had mesenteric metastasis before the operation. The tumors were located in the head of the pancreas in 46 patients and in the body of the pancreas in 16 patients, and the other tumors were located in the tail of the pancreas. In addition, pancreatic ductal adenocarcinoma accounted for 52 of 63 cases, pancreatic ductal adenocarcinoma mixed with mucinous adenocarcinoma accounted for 6 of 63 cases, pancreatic ductal adenocarcinoma mixed with epidermoid carcinoma accounted for 4 of 63 cases, and the others were poorly differentiated adenocarcinomas; 37 patients with pancreatic cancer had lymphatic metastasis.

### 2.3. Immunohistochemistry

**Figure 1:**
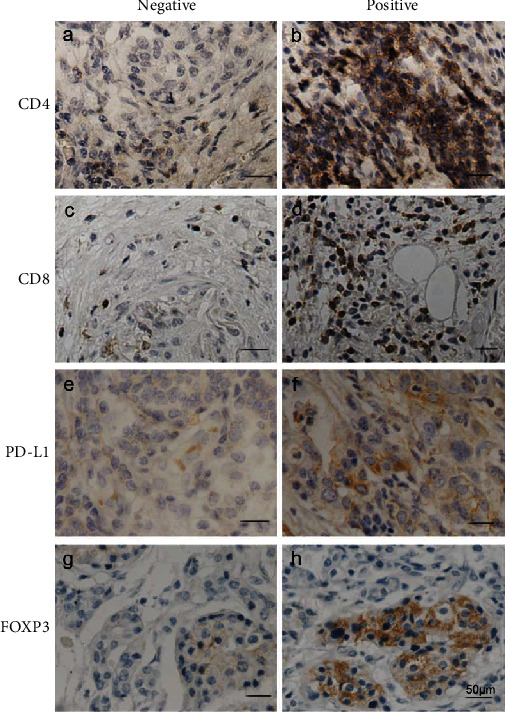
Immunohistochemical staining: (a) negative CD4^+^ T-cell infiltration and (b) positive CD4^+^ T-cell infiltration; (c) negative CD8^+^ T-cell infiltration and (d) positive CD8^+^ T-cell infiltration; (e) negative PD-L1 expression and (f) positive PD-L1 expression; (g) negative FOXP3^+^ Treg cell infiltration and (h) positive FOXP3^+^ Treg cell infiltration. Scale bars represent 50 *μ*m.

Briefly, FFPE specimens were deparaffinized with xylene and rehydrated with a series of decreasing concentrations of ethanol (95%, 90%, 80%, 70%, and 50%) followed by antigen retrieval. After eliminating the endogenous catalase activity, 5% BSA and 10% goat serum were used for blocking. In addition, the specimens were washed with PBS and incubated with primary antibodies at 4°C overnight. The antibodies used were as follows: anti-FOXP3 (ab22510, Abcam) at 1 : 50 dilution, anti-CD4 (ab133616, Abcam) at 1 : 100 dilution, anti-CD8 (ab17147, Abcam) at1 : 100 dilution, and anti-PD-L1 (ab174838, Abcam) at 1 : 100 dilution. The secondary antibodies we used were as follows: polyclonal goat anti-rabbit biotinylated (E0432, Dako), polyclonal rabbit anti-goat biotinylated (E0466, Dako), and ABC Kit (Standard, PK-4000, Vector) and DAB Kit (Standard, SK-4100, Vector). For PD-L1 immunostaining, the cutoff was defined based on the ratio of tumor cells with positive stain to total cells. PD-L1 positivity was defined when the ratio was >10% and PD-L1 negativity when the ratio was <10% [22]. When evaluating CD4^+^ and CD8^+^ T-cells, the number of tumor-infiltrating lymphocytes (TILs) in randomly selected visual fields was calculated. TIL positivity was defined when the number was greater than the average number of TILs. In the cohort of 63 specimens, tumor-infiltrating CD4^+^ and CD8^+^ cells were defined when the number of CD4^+^ and CD8^+^ T-cells was greater than 150 and 100, respectively, in every selected visual field. FOXP3 positivity was defined when the ratio of the positive area to total area was >10% (Figure 1) [23].

### 2.4. Statistics

All data were analyzed using SPSS 19.0 and GraphPad Prism version software. The Kaplan-Meier method was used to estimate the probability of survival, and significance was assessed by the log-rank test. The significance of the difference between PD-L1 expression and FOXP3 expression and several clinical and pathologic variables was assessed by Spearman's rank correlation test. A *P* value < 0.05 was considered statistically significant.

## 3. Results

### 3.1. PD-L1 Expression and FOXP3^+^ Treg Infiltration in Pancreatic Cancer

The PD-L1 expression and FOXP3^+^ Treg infiltration were measured in 63 pancreatic cancer samples using immunohistochemistry. The results showed that 26 patients had positive PD-L1 staining and accounted for 41.3% of cases and 37 patients showed negative PD-L1 staining. Furthermore, 28 patients (44.4%) exhibited high FOXP3^+^ Treg infiltration and 35 patients (55.6%) had low FOXP3^+^ Treg infiltration. In addition, PD-L1 expression levels and FOXP3^+^ Treg infiltration were statistically significantly correlated (*r*^2^ = 0.360, *P* = 0.004).

### 3.2. Correlations among PD-L1 Expression, FOXP3^+^ Treg Infiltration, and Clinicopathologic Characteristics of Pancreatic Cancer

Correlations among PD-L1 expression levels, FOXP3^+^ Treg infiltration, and clinicopathological characteristics of pancreatic cancer patients were investigated. As shown in Table 1, the results indicated that both PD-L1 expression levels and FOXP3^+^ Treg infiltration were statistically significantly associated with depth of invasion (*r*_s_ = 0.472, *P* < 0.001; *r*_s_ = 0.277, *P* = 0.028), lymph node metastasis (*r*_s_ = 0.375, *P* = 0.002; *r*_s_ = 0.483, *P* < 0.001), distant metastasis (*r*_s_ = 0.395, *P* = 0.001; *r*_s_ = 0.274, *P* = 0.03), and pTNM (*r*_s_ = 0.556, *P* < 0.001; *r*_s_ = 0.542, *P* < 0.001), but not associated with age (*r*_s_ = −0.121, *P* = 0.345; *r*_s_ = 0.065, *P* = 0.615) or gender (*r*_s_ = 0.005, *P* = 0.967; *r*_s_ = 0.015, *P* = 0.908).

### 3.3. Associations among PD-L1, FOXP3^+^ Treg Infiltration, and Survival Time

**Figure 2:**
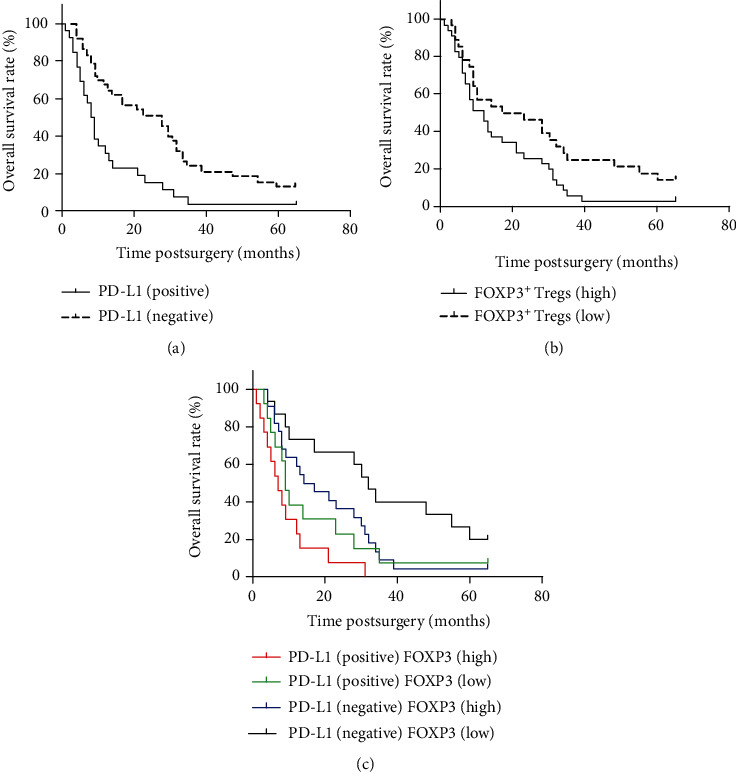
Overall survival rate: (a) overall survival based on PD-L1 expression level, (b) overall survival depending on FOXP3^+^ Treg cell infiltration, and (c) overall survival based on PD-L1 expression level with FOXP3^+^ Treg cell infiltration.

Positive PD-L1 staining was defined when the ratio of the number of tumor cells with positive staining to the total tumor cells was >10%. The survival times of patients with positive PD-L1 staining were compared with those of patients with negative PD-L1 staining; patients with positive PD-L1 staining had worse prognosis (*P* = 0.001). Furthermore, the survival times of patients with high FOXP3^+^ Treg infiltration were compared with those of patients with low FOXP3^+^ Treg infiltration; patients with low FOXP3^+^ Treg infiltration had a better outcome (*P* = 0.009). In addition, all 63 patients were divided into four groups based on PD-L1 expression levels and FOXP3^+^ Treg infiltration, 22 patients (34.9%) showed both positive PD-L1 expression and high FOXP3^+^ Treg infiltration, 6 patients (9.6%) showed positive PD-L1 expression and low FOXP3^+^ Treg infiltration, 15 patients (23.8%) showed negative PD-L1 expression and high FOXP3^+^ Treg infiltration, and 20 patients (31.7%) showed negative PD-L1 expression and low FOXP3^+^ Treg infiltration. The results indicated that the patients with negative PD-L1 expression and low FOXP3^+^ Treg infiltration had the best prognosis among all the four groups and patients in the positive PD-L1 expression and high FOXP3^+^ Treg infiltration group had the worst outcome (Figure 2).

### 3.4. PD-L1 Expression Levels Were Associated with TIL Cells

The relationship between PD-L1 expression levels and TIL cells was investigated. As shown in Table 2, the results indicated a significant negative correlation between PD-L1 expression and tumor-infiltrating CD4^+^ T-cells (*r*_s_ = −0.292, *P* = 0.02) and CD8^+^ T-cells (*r*_s_ = −0.329, *P* = 0.009).

## 4. Discussion

Pancreatic cancer is one of the most common and highly lethal cancers worldwide. Researchers have greatly contributed to the improvement of the survival of patients. Although many novel treatments for pancreatic cancer have recently emerged, none can effectively reduce the mortality of pancreatic cancer [1]. In addition to traditional radiotherapy, chemotherapy, and surgery, immunotherapy might be a potential and effective treatment strategy for pancreatic cancer [24]. Several immunotherapies have already been used clinically, such as antitumor vaccines, which could stimulate the immune system and inhibit tumor growth [25]. However, the efficacy of the vaccines is not satisfactory at present mainly because tumors can escape the host immune system surveillance through various mechanisms [26]. In the present study, PD-L1 expression levels and FOXP3^+^ Treg infiltration were evaluated in a cohort of 63 patients with pancreatic cancer. In addition, the relationships among PD-L1 expression, FOXP3^+^ Treg infiltration, clinicopathologic characteristics, prognosis, and TILs were investigated.

In a previous study, tumor cells were suggested to evade the host immune response which might be due to the following mechanisms: (a) impaired antigen presentation of the tumor cell surface lowers the capacity of T-cells to recognize the decrease in tumor cells, (b) mutation of the major histocompatibility complex (MHC) or antigen-processing genes decreases the capacity of T-cells to recognize tumor cells, and (c) production of immunosuppressive proteins inhibits T-cell activation [27]. These mechanisms allow tumor cells to evade the immune response of patients and then promote tumor cell growth and metastasis [28]. Conversely, stronger immune responses induce activation and aggregation of immune cells and eventually remove the tumor cells in the body [29]. In recent studies, a novel tumor cell escape mechanism was shown that can negatively regulate the immune response through the interaction between PD-1 and PD-L1. PD-L, which is expressed on the surface of tumor cells and antigen-presenting cells, can induce apoptosis of T-cells and promote tumor growth by binding to PD-1 [30]. In addition, TILs were shown in several clinical studies to effectively predict the prognosis in many human cancers [31, 32]. In the present study, the PD-L1 expression level was first measured in pancreatic cancer patients and the expression levels in cancer tissues were statistically negatively correlated with the prognosis of patients. Furthermore, PD-L1 expression levels in pancreatic cancer tissues were associated with invasion depth, lymph node metastasis, distant metastasis, and TNM stage. In addition, PD-L1 expression levels were negatively correlated with CD4^+^ and CD8^+^ T lymphocyte cell infiltration, especially CD8^+^ T lymphocyte cell infiltration. In conclusion, PD-L1 may be a vital factor which can promote tumor cell growth and metastasis in pancreatic cancer.

Blocking the inhibition of the T-cell activation signaling pathway might be a new strategy for cancer treatment in the future [33]. Combination of PD-1/PD-L1 pathway inhibitors and traditional anticancer drugs will be more effective in cancer treatment [34, 35]. In previous studies, PD-L1 expression levels were significantly associated with distant metastasis and survival time of patients [36]. In the present study, PD-L1 expression levels were confirmed to be significantly associated with prognosis, invasion depth, lymph node metastasis, distant metastasis, and lymphocyte infiltration. These prognostic biomarkers will play a crucial role in the process of exploring anticancer strategies. Additional clinical data on pancreatic cancer are necessary to determine the clinical significance of PD-L1.

FOXP3, which is a common immunohistochemical marker for Tregs, has been confirmed to be closely associated with the prognosis of patients with cancer [37]. In a previous study, the prognostic value of FOXP3^+^ Tregs in different gastrointestinal cancers remains controversial. In a recent meta-analysis, FOXP3^+^ Treg infiltration was found to be associated with poor prognosis in hepatocellular cancer and gastric cancer patients and good prognosis in CRC patients [38]. In a previous study, FOXP3^+^ Treg infiltration was reportedly associated with poor prognosis in pancreatic cancer patients [39].

Lymph node metastasis and distant metastasis are important for a variety of cancers, especially pancreatic cancer [40]. Lymph nodes are important for antigen presentation and immune response and act as a barrier to prevent tumor cells from entering the circulatory system [41, 42]. The local microenvironment of lymph nodes is vital to the immune response, and occurrence of distant metastasis is associated with patient prognosis [43, 44].

In the present study, the relationship between FOXP3^+^ Tregs and lymph node metastasis and distant metastasis in 63 patients with pancreatic cancer was analyzed and a significant correlation with survival time, lymph node metastasis, and distant metastasis was observed. Deng et al. confirmed similar results in CRC [45]. In the present study, results indicated that FOXP3^+^ Treg-mediated immune tolerance promotes cancer cell proliferation and metastasis. FOXP3^+^ Treg infiltration in pancreatic cancer patients was evaluated and negatively correlated with prognosis. FOXP3^+^ Treg infiltration was also associated with invasion depth, lymph node metastasis, distant metastasis, and TNM stage. Chemotactic factors, cell metabolism, oxidative stress, and pH in the tumor microenvironment could affect FOXP3^+^ Treg infiltration in cancer tissues [39]. Moreover, the underlying mechanisms of Treg infiltration in pancreatic cancer should be further studied.

## 5. Conclusion

In conclusion, our study showed that PD-L1 and FOXP3 are prognostic biomarkers for pancreatic cancer. PD-L1 expression levels and FOXP3^+^ Treg infiltration are correlated with prognosis of patients with pancreatic cancer. Moreover, PD-L1 expression level and FOXP3^+^ Treg infiltration correlated with lymph node metastasis, distant metastasis, and TNM stage. The expression level of PD-L1 significantly correlated with infiltration of FOXP3^+^ Treg and negatively correlated with TIL infiltration.

## Figures and Tables

**Table 1:** Correlations among PD-L1 expression, FOXP3^+^ Treg infiltration, and clinicopathologic characteristics of pancreatic cancer patients.

PD-L1 expression					FOXP3^+^ Treg infiltration			
Clinicopathological parameters	Negative *n* (%)	Positive *n* (%)	*r*_s_	*P* ^∗^	Negative *n* (%)	Positive *n* (%)	*r*_s_	*P* ^∗^
No. of patients	37 (58.7)	26 (41.3)			28 (44.4)	35 (55.6)		
Age (years)								
<65	14 (51.6)	13 (48.1)	-0.121	0.345	13 (48.1)	14 (51.9)	0.065	0.615
≥65	23 (63.9)	13 (36.1)			15 (41.9)	21 (58.3)		
Gender								
Female	13 (59.1)	9 (40.9)	0.005	0.967	10 (45.5)	12 (54.5)	0.015	0.908
Male	24 (58.5)	17 (41.5)			18 (43.9)	23 (56.1)		
Tumor status								
T1	5 (100)	0 (0)	0.472	<0.001^∗∗∗^	4 (80)	1 (20)	0.277	0.028^∗^
T2	14 (87.5)	2 (12.5)			9 (56.3)	7 (43.7)		
T3	18 (43.9)	23 (56.1)			15 (36.6)	26 (63.4)		
T4	0 (0)	1 (100)			0 (0)	1 (100)		
Nodal status								
N0	21 (80.8)	5 (19.2)	0.375	0.002^∗∗^	19 (73.1)	7 (26.9)	0.483	<0.001^∗∗∗^
N1	16 (43.2)	21 (56.8)			9 (56.8)	28 (56.8)		
Metastatic status								
M0	36 (66.7)	18 (33.3)	0.395	0.001^∗∗^	27 (50)	27 (50)	0.274	0.03^∗^
M1	1 (11.1)	8 (88.9)			1 (11.1)	8 (88.9)		
Pathologic status								
Stage I	8 (88.9)	1 (11.1)	0.556	<0.001^∗∗∗^	7 (77.8)	2 (22.2)	0.542	<0.001^∗∗∗^
Stage IIa	19 (82.6)	4 (17.4)			16 (69.6)	7 (30.4)		
Stage IIb	9 (42.9)	12 (57.1)			4 (19)	17 (81)		
Stage III	0 (0)	1 (100)			0 (0)	1 (100)		
Stage IV	1 (11.1)	8 (88.9)			1 (11.1)	8 (88.9)		
FOXP3^+^ Treg infiltration								
Low	22 (78.6)	6 (21.4)	0.360	0.004^∗∗^				
High	15 (42.9)	20 (57.1)						

**Table 2:** The PD-L1 expression levels were associated with tumor-infiltrating lymphocyte (TIL) cells.

	PD-L1 expression		*r*_s_	*P* ^∗^
	Positive	Negative		
CD4 (%)				
Positive	14 (53.8)	30 (81.1)	-0.292	0.02^∗^
Negative	12 (46.2)	7 (18.9)		
CD8 (%)				
Positive	13 (50.0)	30 (81.1)	-0.329	0.009^∗∗^
Negative	13 (50.0)	7 (18.9)		

## Data Availability

All data used to support the findings of this study are available upon request to the corresponding author.
